# Social Functioning in Chinese College Students with and without Schizotypal Personality Traits: An Exploratory Study of the Chinese Version of the First Episode Social Functioning Scale

**DOI:** 10.1371/journal.pone.0061115

**Published:** 2013-05-14

**Authors:** Yi Wang, Ya-hsuan Yeh, Sin-man Tsang, Wen-hua Liu, Hai-song Shi, Zhi Li, Yan-fang Shi, Ya Wang, Yu-na Wang, Simon S. Y. Lui, David L. Neumann, David H. K. Shum, Raymond C. K. Chan

**Affiliations:** 1 Neuropsychology and Applied Cognitive Neuroscience Laboratory, Key Laboratory of Mental Health, Institute of Psychology, Chinese Academy of Sciences, Beijing, China; 2 The University of Chinese Academy of Sciences, Beijing, China; 3 Department of Applied Social Studies, City University of Hong Kong, Hong Kong; 4 Faculty of Humanities and Social Sciences, Guangzhou Medical College, Guangzhou, China; 5 School of Applied Psychology, Griffith University, Gold Coast, Australia; 6 Behavioural Basis of Health Program, Griffith Health Institute, Gold Coast, Australia; Catholic University of Sacred Heart of Rome, Italy

## Abstract

**Objectives:**

The First Episode Social Functioning Scale (FESFS) was designed to measure social functioning of young individuals with schizophrenia. The aim of this study was to validate a Chinese version of the FESFS in a sample of young Chinese adults.

**Method:**

The FESFS was translated to Chinese prior to being administered to 1576 college students. The factor structure, reliability, and validity of the scale were examined.

**Results:**

Two items were deleted after item analysis and the internal consistency of the whole scale was .89. A six-factor structure was derived by exploratory factor analysis. The factors were interpersonal, family and friends, school, living skills, intimacy, and balance. Estimates of the structural equation model supported this structure, with Goodness of Fit Chi-Square χ^2^ = 1097.53 (p<0.0001), the root mean square error of approximation (RMSEA) = 0.058, and the comparative fit index (CFI) = 0.93. Scale validity was supported by significant correlations between social functioning factors scores and schizophrenia personality questionnaire (SPQ) scores. Individuals with schizotypal personality features presented poorer social functioning than those without schizotypal personality features.

**Conclusions:**

The Chinese revised version of the FESFS was found to have good psychometric properties and could be used in the future to examine social functioning in Chinese college students.

## Introduction

Social functioning is one of the key outcome variables in psychological research. It is also a major deficit across a range of psychotic disorders [1]–[4]. Social functioning is a broad construct that reflects an overall performance across many everyday domains (e.g., independent living, employment, interpersonal relationships, and recreation) [5], [6]. The impact of social functioning is observed across different life stages, such as learning at school, working in a job, and even during retirement. An individual's social environment and interactions with others is constantly changing, which causes people to adapt to different social role and activities. Thus, when measuring social functioning, especially deficits in social functioning, it is important to use a measure that reflects a person's actual and optimal functioning and corresponds to his/her age-appropriate social activities.

There are some clinical interview based measurements for social functioning. For example, the Quality of Life Scale (QLS) [7] is a 21-item semi-structured interview designed to rate deficit symptoms. However, there are a limited number of self-report measurements for social functioning that are suitable for clinical patients [8]. Based on the Client's Assessment of Strengths, Interests, and Goals (CASIG), [9] used in clinical process of planning and evaluating treatment, Lecome et al. developed the First Episode Social Functioning Scale (FESFS) [10] to capture the different domains of community functioning skills relevant for people with early psychosis. Unlike the structured interview assessment used in the CASIG, the FESFS is a self-reported questionnaire. It aims to measure several domains of everyday functioning such as living skills, interacting with people, social activities, intimacy, friends, family, work and school.

Although the FESFS was originally designed to capture the social functioning of individuals following a first-onset of psychosis, the items of the scale can reflect the overall social functioning of individuals with similar though perhaps attenuated social behaviours such as those seen in individuals with schizotypal personality features. Moreover, the scale can also measure social functioning in non-psychotic or non-schizotypal individuals. The main aim of the current study was to validate the Chinese version of the FESFS in a non-clinical college student sample. In addition, we measured schizotypal personality features in the sample. Recent studies have found that people with schizotypal personality features demonstrate social functioning impairments [11]–[13]. We thus hypothesized that students with schizotypal personality features would demonstrate significantly poorer functioning than those without schizotypal personality features.

## Methods

### Participants

One thousand seven hundred and ninety seven college students were recruited from three cities in China (Beijing, Shanghai, and Guangzhou). All participants were first-year college students, whose first language were Chinese and majored in education, medicine and economics. The participants completed the set of questionnaires individually in groups. We excluded the cases with any missing data on age, gender and items of social functioning scale A- questions (the items we used for the revision of Chinese version). The final sample consisted of 1576 participants (594 males; mean age: 18.8 years, SD = 0.8; mean years of education: 12.30 years, SD = 0.66). In terms of ethnicity, 1499 (95.1%) students were Han Chinese and the others were minority. In terms of living place before 12 years old, 745 students (47.3%) reported that they lived in rural areas, 757 students (48.0%) lived in cities, and 74 students did not report this information.

### Ethics Statement

The study was approved by the ethics committee of the Institute of Psychology, Chinese Academy of Sciences. Written informed consent was obtained from each participant before the study began.

### Measures

#### Chinese Version of the First Episode Social Functioning Scale

The original English version FESFS [10] is a comprehensive rating scale designed to assess the social functioning of individuals with early psychosis. The scale was designed to measure eight domains of social functioning, namely: Living Skills, Interacting with People, Social Activities, Intimacy, Friends, Family, Work and School. For each domain, three to seven facets are investigated with two types of questions: A- assesses the individual's perceived ability (e.g., “I find it easy to interact with authority figures”); B- assesses the individual's actual performance of the behavior (e.g., “In the past 3 months, I have been interacting with authority figures”). Each question is evaluated on a four-point Likert scale (ranging from 1 = totally disagree to 4 = totally agree for question type A, and from 1 = never to 4 = always for question type B), with specific probes and examples for each item depending on the area assessed. Originally, the FESFS has two versions: the individual self-report version and the clinician's informant-report. Both versions include the same items, but are phrased in the first or in the third person depending on the version.

The Chinese version of the FESFS for young adults was reconstructed based on the items from the FESFS. We approached the author of the original version of the FESFS for her approval of validating the checklist in mainland China. We only adopted 34 A type questions from the FESFS. These questions assessed an individual's perceived social functioning ability from the FESFS. In developing the Chinese version of the FESFS, we followed the international guidelines for cross-cultural validation of self-reported measures [14]. The five steps for a proper cross-cultural validation were: (1) translation of the original checklist into the to be used language, Chinese in this instance; (2) synthesis of the translations; (3) back-translation of the to be used language to the original language, English in this instance; (4) expert panel review on the relevancy and representation of items used for the setting, mainland China in this situation; and (5) pilot testing of the validated checklist. The instrument items were translated into Chinese by two of the authors (one psychology postgraduate YFS and one psychiatrist SSYL). The items were then passed onto one bilingual colleague (YW) for back translation to English. We also obtained feedback from the original author on the back-translated version of the scale. The original and the back-translated versions were compared by the senior author (RCKC), and any inconsistencies were discussed and resolved by modifying the Chinese version. The translated items were then examined by a panel of four members, including a neuropsychologist specialized in psychometrics (RCKC), a psychiatrist (SSYL), a social psychologist (WHL), and a psychology postgraduate student (YFS). Members of the panel determined whether the items were relevant and representative of the Chinese setting. If necessary, the items would be changed or deleted to better represent the Chinese cultural context. In actual fact, the panel found all the items to be culturally appropriate in the Chinese context and did not make many changes when translating them into Chinese. The translated FESFS was then administered to thirty college students for pilot testing and they were asked if the terms were clear and easy to understand s. The final self-report version was then administered to all the participants of the current study.

#### Schizotypal Personality Questionnaire, SPQ

The Schizotypal Personality Questionnaire (SPQ) [15] is a self-report scale modeled on the DSM-III-R criteria for schizotypal personality disorder that contains subscales for all nine schizotypal traits. It is used to screen for schizotypal personality disorder in the general population. Participants respond YES or NO to all 74 questions related to “idea of reference”, “excessive social anxiety”, “odd beliefs”, “unusual perceptual experiences”, “odd behavior/speech”, “suspiciousness”, “constricted affect”, and “no-close-friends”. We used the Chinese version of the SPQ[16], which has been shown to have satisfactory reliability and validity. The reliability of the Chinese version SPQ as measured by Cronbach's alpha was 0.91 for the whole scale and 0.71–0.78 for its three subscales [16].

### Data analysis

Initially, we checked the distribution of responses for each item and confirmed that there was no skewness present. The original FESFS contains items related to an individual's work. However, because the present sample consisted of college students and most of them do not work, the items related to work were excluded prior to data analysis. For the remaining items, item analysis and Cronbach's alpha analysis was conducted using SPSS 16.0. Any items that showed lower than 0.30 on item-total correlations were removed. Next, the data set was randomly split into two sample pools. For the first half of the sample (Sample A, n = 761), principal component analysis with varimax rotation was conducted to explore the underlying factors of the scale. Factors with eigenvalues higher than 1.0 were retained. For the second half of the sample (Sample B, n = 815), we performed a confirmatory factor analysis using LISREL 8.7 to verify the model resulting from the first sample.

To examine the validity of the scale, we conducted Pearson correlation analysis between SPQ scores and FESFS scores using data obtain in Sample B. In addition, individuals with and without SPD features (screened by SPQ scores) were compared on social functioning performance.

## Results

### Reliability and item analysis

First of all, the distribution of each item was checked and no item showed extreme skewness. Two items, “I am really good in solo activities that are not simply watching TV, listening to music or playing videogames, such as going to the gym, going to the movies, chatting on the net, taking lessons (music, painting, etc)” and “I am interested in sex”, were removed, because they had a corrected item-total correlation lower than .30. After deletion of these two items, the internal consistency of the scale as measured by Cronbach's coefficient alpha was.89. The total number of items thus was reduced from 34 to 32.

### Exploratory factor analysis

An exploratory factor analysis with principal component and varimax rotation was carried out on all 32 items (KMO = 0.909, Chi-square = 6562, p<0.0001). According to the scree plot, 6 factors could be extracted, 5 items were deleted because of the high loadings on more than one factor (the five items were listed at the note of Table 1). We then rerun the exploratory factor analysis with 27 items, the total variance explained by the 6 factors was 47.87%. As shown in Table 1, the factors were labeled as: interpersonal, family and friends, school, living skills, intimacy, and balance. The Cronbach's coefficient alpha for each factor were .75, .68, .73, .54, .61, and .56 respectively.

**Table 1:** Rotated factor loading of each item following principle components analysis.

Item	A	B	C	D	E	F
1. I find it easy to interact with authority figures (e.g. teacher, boss, doctor, others' parents…)	0.700					
2. I find it easy to talk with people my age I know just a little bit	0.658					
3. I am able to make new friends by suggesting getting together, making invitations or phoning people up	0.613					
4. I am good at resolving conflicts between people	0.598					
5. I find it easy to interact with waiters, cashiers, and salespeople (e.g. small talk, asking for information, making a purchase)	0.544			0.321		
6. I participate well in extra-curricular group activities such as group sports, organizations, church, and clubs	0.538		0.318			
7. I can quickly understand what is going on in most situations involving other people	0.502					
8. My parents and I typically get along		0.684				
9. I feel I have at least one best friend with whom I can share important things that happen to me		0.618				
10. I can talk to my parents about things that matter to me		0.608				
11. I have friends that I can hang out with, do stuff with (shopping, movies, go out…)		0.554		0.341		
12. My brothers/sisters and I typically get along		0.549				0.325
13. I am able to consistently get good grades			0.697			
14. I am always able to finish my assignments on time			0.690			
15. I come to the school/college/university on time and rarely miss classes			0.624			
16. I take steps at school/college/university to meet my educational goals (go to library, meet with teachers, etc)			0.557			
17. I am comfortable participating in the classroom			0.445			
18. I am comfortable using the phone, internet or email to communicate				0.683		
19. I have no problem getting enough food to eat (by cooking, family, fast food, etc)				0.678		
20. I can get around town easily, either by taking the bus or by other means of transportation.				0.469		
21. I am good at taking care of my physical appearance and hygiene				0.459		
22. I enjoy having a stable boy/girlfriend or spouse					0.800	
23. I am quite comfortable dating					0.687	
24. I feel I am able to share feelings, inner thoughts, and be close with my stable boy/girlfriend or spouse (when I have one)		0.358			0.639	
25. I am good at handling money (budgeting, not spending it all at once)						0.701
26. I do my household tasks well (e.g. washing dishes, cleaning my room, vacuuming)						0.648
27. I am able to balance the amount of time I spend with others and by myself	0.310					0.427

Factors: A – Interpersonal; B- Family and Friends; C – School; D – Living Skills; E – Intimacy; F – Balance. Those five items deleted because of high loading on more than one factor were: “I know how to stand up for myself when needed”, “I try to do things that are really important to me (specific hobbies, passions…)”, “I get along well with my extended family (grandparents, uncles, aunts, cousins)”, “I am able to talk to my teacher/professor about things at school/college/university that matter to me (classes, assignments, schedules, etc.)”, “The other students and I typically get along”.

### Confirmatory factor analysis

**Figure 1:**
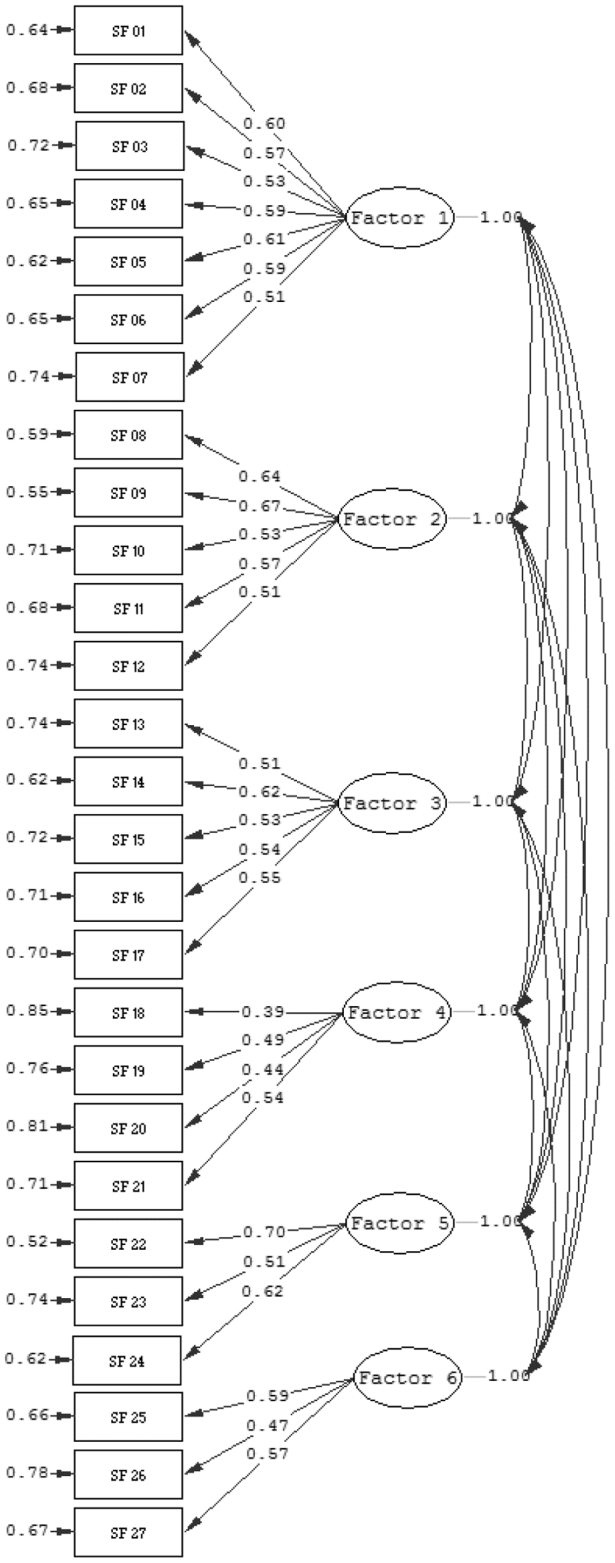
The structure of social functioning scale by structural equation modeling analysis. Estimates of the structural equation model supported the six factor structure of the social functioning scale, with Goodness of Fit Chi-Square χ^2^ = 1097.53 (p<0.0001). The Root Mean Square Error of Approximation (RMSEA) = 0.058, RMSEA 90% CI = (0.054, 0.062), Comparative Fit Index (CFI) = 0.93, and Standardized Root Mean Square Residual (SRMR) = 0.054. Factor names: Factor 1: interpersonal; Factor 2: family and friends; Factor 3: School; Factor 4: Living skills; Factor 5: Intimacy; Factor 6: Balance.

Confirmatory factor analysis was used to test the six-factor model. Figure 1 shows the result of the confirmatory factor analysis in which a six-factor solution was supported. Minimum fit function chi-square was 1097.53, with 309 degrees of freedom. The model was assessed using the comparative fit index (CFI), the root mean square error of approximation (RMSEA), and the standardized root mean square residual (SRMR) as recommended by Kline [17]. CFI values greater than or equal to 0.90 [18] and RMSEA smaller than 0.06 indicate good model fit [19]. For SRMR, values less than 0.08 are regarded as having acceptable fit. The CFI and RMSEA values suggest the model fits well with the data (CFI = 0.93, RMSEA = 0.058). In addition, the SRMR was acceptably low (SRMR = 0.054). Taken together, results of the the confirmatory factor analysis indicated that the six-factor model fitted the data well. The six factors are interpersonal, family and friends, school, living skills, intimacy, and balance.

### Social functioning and schizotypal personality features

Table 2 shows the correlations between the derived factor scores of the FESFS and SPQ. SPQ total score correlated significantly with social functioning factors, except for family and friend. Higher total SPQ scores were associated with lower interpersonal (r = −0.34, p<0.01), school (r = −0.20, p<0.01), living skills (r = −0.14, p<0.01), intimacy (r = −0.08, p<0.05) and balance (r = −0.17, p<0.01) scores. We found significant negative correlations between SPQ interpersonal and social functioning interpersonal subscale (r = −0.50, p<0.01) scores. Besides, considering the subscales of SPQ, there were significant negative correlations between interpersonal SPQ scores and all social functioning factors. Higher cognitive-perceptual SPQ scores were associated with lower interpersonal, school, and balance factor scores. Higher disorganized SPQ scores were correlated with lower interpersonal, school, living skills, and balance factor scores.

**Table 2:** Correlations between SPQ and factors of social functioning.

	SF_Interpersonal	SF_Family and friends	SF_School	SF_Living skills	SF_Intimacy	SF_Balance
SPQ_cognitive perceptual	−.100*	0.031	−.090*	−0.014	0.005	−.104**
SPQ_interpersonal	−.495**	−.185**	−.280**	−.224**	−.166**	−.194**
SPQ_disorganized	−.197**	−0.029	−.134**	−.083*	−0.032	−.142**
SPQ_total	−.342**	−0.074	−.202**	−.138**	−.081*	−.172**

SF = First Episode Social Functioning Scale; SPQ = Schizotypal Personality Questionnaire.

* : p<0.05;

** : p<0.01.

According to the total score of the SPQ, 66 participants were classified as individuals with schizotypal personality features (top 10th percentile on SPQ total scores) and 70 were classified as without schizotypal personality features (bottom 10% percentile on SPQ total score). As shown in Table 3, the two groups differed significantly on all but the family and friends factor (p = 0.117) of the Chinese version of the FESFS for young adults.

**Table 3:** Comparison between individuals with and without schizotypal personality features on social functioning scale.

	non-SPD (n = 66)		SPD (n = 70)			
	Mean	S.D.	Mean	S.D.	t	p
SF_Interpersonal	2.87	0.37	2.53	0.40	5.15	<0.001
SF_Family and friends	3.24	0.37	3.13	0.40	1.58	= 0.117
SF_School	3.12	0.32	2.93	0.36	3.20	<0.01
SF_Living skills	3.14	0.35	2.93	0.40	3.27	<0.01
SF_Intimacy	3.12	0.47	2.92	0.50	2.38	<0.05
SF_Balance	2.82	0.35	2.67	0.45	2.20	<0.05

## Discussion

The present results showed that the Chinese version of social functioning scale reconstructed from the FESFS had good reliability and construct validity in a Chinese sample of young adults. Results of exploratory and confirmatory factor analysis showed that there is good fit for a six factor solution, including “interpersonal”, “school”, “living skills”, “intimacy”, “family and friends” and “balance”. The scale showed good internal consistency and high convergent validity. The SPQ total score correlated significantly with the social functioning factors. In addition, individuals with SPD features showed lower social functioning scores than those without SPD features.

The six factors derived from exploratory factor analysis were labeled as interpersonal interaction (Cronbach's alpha was 0.75); family and friends (0.68); school (0.73); living skills (0.54); intimacy (0.61), and balance (0.56). Seven items comprise the interpersonal interaction factor, asking participants to assess their own behavior on the interaction with other people or in social activities. The family and friends factor include five items, which describe an individual's social interaction with parents or friends. There are five items relating to the performance in school, and four items relating to living skills; while three items from intimacy focused on the relationship with boy/girlfriend. The other three items relate to the balance, including how to spend money, time and do housework. The internal consistency coefficients for three factors, “balance”, “intimacy” and “living skills”, are lower than expected. One of the reasons might be the small number of items in these factors. In future studies we will include more items in these scales to improve their internal consistencies.

Although there are some other measurements for social functioning, such as the semi-structure interview based QLS, the FESFS has its own advantage. That is, the FESFS can be used to assess a wide range of populations, from college students to adult, normal people to first episode psychotic patients. The multi-dimensional nature of the scale allows us to assess social functioning more comprehensively. The six factor structure of the Chinese version of the FESFS showed a good fit to the model. The main indices of RMSEA and CFI supported the construct validity of the scale. These six factors represent different facets of college social life in China. There is one interesting factor found in our current findings, that is, the factor “balance”, which assesses the balance of money, time and housework of college students. For college students in China, most of them first left their parents and started to take care of themselves when they started university. They have to learn how to manage their expenses and time is also an important thing for them. Therefore, we have kept this factor and would revise it by adding more items in the future. Because all participants were college students and most of them do not have to work, items related to work were excluded. Work-related items may be relevant for adults in the general population but not for students. This represents a potential limitation of the present study. Nevertheless, given that the aim of this study was to validate the social functioning scale in Chinese college students and to make the scale available for use with these students, the exclusion of work-related items is reasonable. Future research should apply the scale to a sample of young adults in the general population who work to validate the work-related scale. More importantly, given the nature of the original scale, we have to validate the scale in clinical patients with first-episode psychosis and high-risk individuals prone for psychosis.

The SPQ measures an interpersonal factor that includes the traits of “excessive social anxiety”, “no close friends”, “suspiciousness” and “constricted affect” [15]. This factor was found to be strongly and negatively associated with scores on the social functioning scale, especially for the factor of interpersonal interaction. Further, when we compared individuals with and without schizotypal personality features, significant differences were found between the two groups on all but one of the social functioning factors. These results are consistent with a previous study that showed schizotypy traits were negatively correlated with social functioning as measured by the Social Functioning Scale [12]. It is also consistent with studies which showed that participants with high schizotypy have significantly impaired academic performance as measured by Social Adjustment Scale-Self Reported [11], [13]. However, the present results are not consistent with previous findings that higher schizotypy individuals show impairments in their relationships with peers and family. This difference might reflect that the FESFS was more comprehensive and some items related to relationships with peers and family were extracted to other factors such as interpersonal or intimacy, and these domains were impaired in our present SPD group.

Altogether, the present results indicated good reliability, construct validity, criterion-related validation of our reconstructed Chinese version social functioning scale. In order to adopt this scale in Chinese populations, including both individuals with schizotypal personality features and first episode patients with schizophrenia, future studies need to be carried out to include additional items for factors that have few items, add work-related items for the work factor, and validate the scale in schizophrenia patients.
